# Analytic Design of Segmented Phase Grating for Optical Sensing in High-Precision Alignment System

**DOI:** 10.3390/s21113805

**Published:** 2021-05-31

**Authors:** Guanghua Yang, Jing Li, Yu Wang, Minxia Ding, Lina Zhong

**Affiliations:** 1 Institute of Microelectronics, Chinese Academy of Sciences, Beijing 100029, China; yangguanghua@ime.ac.cn (G.Y.); ywang@ime.ac.cn (Y.W.); dingminxia@ime.ac.cn (M.D.); zhonglina@ime.ac.cn (L.Z.); 2 University of Chinese Academy of Sciences, Beijing 100049, China

**Keywords:** phase grating, alignment system, diffraction efficiency

## Abstract

Ultra-precision measurement systems are important for semiconductor manufacturing processes. In a phase grating sensing alignment (PGA) system, the measurement accuracy largely depends on the intensity of the diffraction signal and its signal-to-noise ratio (SNR), both of which are associated with the grating structure. Although an equally segmented grating structure could increase the signal of a high odd order, it could also strengthen the signals at the zeroth and even orders which are the main contributors of stray light. This paper focuses on the practical problem of differently responding diffraction orders but in one grating structure. An analytical relationship has been established between the diffraction efficiency and the segment structure of phase grating. According to this analytic model, we then propose a design method to increase the diffraction signal at high odd orders and, meanwhile, to decrease it at the zeroth and even orders. The proposed method provides a fast and effective way to obtain the globally optimal grating structure in the valid scope. Furthermore, the design examples are also verified by means of numerical simulation tool–rigorous coupled-wave analysis (RCWA) software. As a result, the proposed method gives insight into the diffraction theory of segmented grating and the practical value to greatly improve the design efficiency.

## 1. Introduction

Ultra-precision measurement systems play an essential role in cutting-edge technology and industrial process quality control [1]. In semiconductor manufacturing, an alignment sensing system has been applied to measure wafer positions, which is a prerequisite to meet the stringent overlay budget [2,3,4,5]. One type of alignment sensing system uses optical microscopy to illuminate the target, e.g., grating, with broadband spatial incoherent light. The magnified image of grating is captured by a CCD or CMOS array detector [6]. Then, the exact location is analyzed by image processing algorithms. However, a few challenges will be encountered when applying it in practice. One of the big challenges is the negative impact of lens aberration [7,8]. Position error caused by lens aberration often accumulates to 1 nm which consumes a large part of the 4 nm overlay requirements of today’s alignment [5]. This kind of problem can be effectively solved by the other type of alignment sensing system, i.e., phase grating alignment (PGA). The principle of PGA is that incident light irradiates uniformly at the grating surface and produces diffraction light with different orders. The targeted position can be extracted by the phases of the diffraction signals [9,10].

In general, the PGA sensors can detect nine diffraction orders of phase grating [9,10]. The diffraction light of the odd orders (DLO) is useful to calculate the measurement positions. However, the diffraction light of the zeroth and the even orders (DLZE) is the main contributor of stray light which greatly deteriorates the signal quality. The diffraction light of the lower odd orders (L-DLO), such as the 1st and the 3rd, are often used to determine the measurement range, while the diffraction light of the higher odd orders (H-DLO), such as the 5th, the 7th, and the 9th, are often used to improve measurement accuracy [10]. In practice, the intensity of H-DLO is usually very low [11,12]. For a typical integrated circuit chip (IC) manufacturing process, deformation of phase grating happens at many steps, such as etching, polishing, and deposition, and thus will further reduce the intensity of H-DLO, finally leading to wrong positioning and product failures [11,12]. Besides this, DLZE should be restrained in order to increase the signal-to-noise ratio (SNR) of the measurement signal [12]. Although the unwanted DLZE can be polarized or covered, it is still hard to eliminate completely. Therefore, designing a grating structure with enhanced H-DLO and reduced DLZE at the same time is an interesting challenge for alignment sensing systems used in the IC industry.

The common basic period of phase grating is 16 μm in a PGA system [5]. For a phase grating with a duty cycle of 0.5, the diffraction efficiency decreases while the order number increases [5]. In this case, the diffraction efficiency supported by the 5th order light signal is rather inadequate for a robust positioning. A segmented phase grating structure is designed to improve the light signals received at high odd orders [13,14,15,16,17]. For the 5th order, the grating ridge is further equally divided into five parts, as three ridges and two grooves, which is labeled as AH53 by taking the naming rule in [15] (p. 854). Likewise, AH74 is defined with seven equally segmented parts, including four ridges and three grooves, to improve the 7th order. Unfortunately, these equally segmented designs not only enhance the efficiency of the H-DLO but also magnify the efficiency of the DLZE. To avoid this, numerical simulation has been performed by using commercial software tools such as COMOSL to search for better structure parameters [18,19,20,21,22,23,24]. However, it is still very hard to find the overall optimal solution because the numerical method is quite sensitive to the initial conditions. Furthermore, tuning parameters numerically is a lengthy and costly process that requires a high-powered processor to deal with multiple variables simultaneously. The simulation complexity will be further increased and hence the design efficiency drops dramatically when multi-wavelength illumination is implemented to improve the physical signal robustness and to reduce the impact of asymmetric grating distortion, e.g., in some alignment systems using 532 nm and 633 nm wavelengths [5], some using 532 nm, 633 nm, 780 nm, and 852 nm wavelengths [10], and some even using a white light source [25]. A lot of research has been done on phase gratings based on scalar diffraction theory [26,27,28], especially in the field of Dammann grating [29,30]. A Dammann grating is a pure phase modulation grating in which phase transition points are optimized to produce equal-intensity spots at diffractive orders with high efficiency [29]. To realize even-numbered spot arrays, Morrison introduced a translation symmetry method for the effect with all the even-number spectra suppressed [31,32], which could be further applied to an alignment sensor system with all the even orders eliminated.

In this paper, we propose a design method for a phase grating structure based on scalar diffraction theory. This method provides a fast and reliable way to find the most effective strategy that can tackle the practical problems of both H-DLO and DLZE at the same time. Firstly, we construct an analytic model to describe the relationship between the diffraction efficiency and the structure parameters of segmented phase grating. Based on the relationship, a design strategy of grating structures is then developed with diffraction efficiency of the H-DLO increased and that of the DLZE decreased. In order to apply the design in the practical case of the IC industry, multi-wavelength illumination, 532 nm and 633 nm, was chosen to illustrate the effect. By this method, we easily obtain alternative design options with the 5th order specifically enhanced or the 7th order specifically enhanced. Finally, the design results are validated and compared with the numerical software tool RCWA.

## 2. Analytical Model

### 2.1. Diffraction Efficiency of Standard Phase Grating

**Figure 1:**
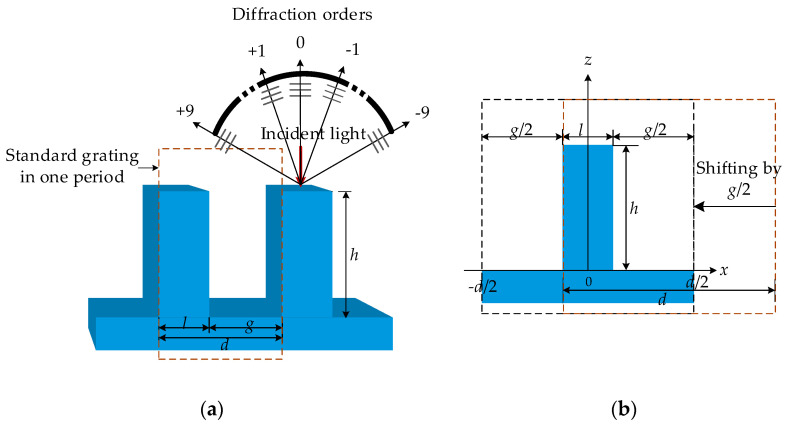
(**a**) The profile of a standard phase grating; (**b**) a cross-section in one period.

Here, one ridge in one period is called a standard phase grating. Figure 1 illustrates the profile of a standard phase grating in 3D and a cross-section case at the *x-z* plane by shifting a standard one. In Figure 1, *d* is the period length, *l* is the ridge width, *g* is the groove width, *h* is the groove depth, the duty cycle *f* is defined as *f* = *l*/*d*, and *w* is defined as *w* = *g*/*d.*

Suppose a grating structure extends infinitely along the *y*-axis and has an infinite number of periods along the *x*-axis. In the coordinate system defined in Figure 1, the profile of the standard phase grating is described by the function *z*(*x*):

(1) $$z\left(x\right)=\{\begin{array}{ll} 0, & \mathrm{if}\;fd/2+nd<\left|x\right|\leq d/2+nd\\ h, & \mathrm{if}\;\left|x\right|\leq fd/2+nd \end{array}$$

where *n* is non-negative integers. The structure shown by the function *z*(*x*) represents a phase grating. Hence, its reflection function *R*(*x*) is represented as follows:

(2) $$R\left(x\right)=\{\begin{array}{ll} r, & \mathrm{if}\;fd/2+nd<\left|x\right|\leq d/2+nd\\ r\exp\left(-j4\pi h/\lambda \right), & \mathrm{if}\;\left|x\right|\leq fd/2+nd\; \end{array}$$

where *r* is the reflection coefficient of the grating material. It depends on the grating material and wavelength, assuming that the incident light is a plane wave with amplitude *A*_0_ = 1 and is perpendicular to the grating surface. Based on the scalar diffraction theory, the diffraction field of the *m*^th^ order is then defined as:

(3) $$U_{m}=\frac{A_{0}}{d}\int_{-d/2}^{d/2}R\left(x\right)\exp\left(-j\frac{2m\pi }{d}x\right)dx,$$

Substituting Equation (2) into Equation (3), the diffraction field *U_m_* would be:

(4) $$U_{m}=\{\begin{array}{ll} r\left\{1+f\left[\exp\left(-j\frac{4\pi h}{\lambda }\right)-1\right]\right\}, & \mathrm{if}\;m=0\\ r\frac{\sin\left(m\pi f\right)}{m\pi }\left[\exp\left(-j\frac{4\pi h}{\lambda }\right)-1\right], & \mathrm{if}\;m=\pm 1,\pm 2\cdots \; \end{array}$$

According to Equation (4), the diffraction efficiency *η_m_* of standard phase grating is obtained:

(5) $$\eta_{m}=\frac{{\left|U_{m}\right|}^{2}}{{\left|A_{0}\right|}^{2}}=\{\begin{array}{ll} r^{2}\left[1-4f\left(1-f\right)\sin^{2}\left(\frac{2\pi h}{\lambda }\right)\right], & \mathrm{if}\;m=0\\ r^{2}\frac{4\sin^{2}\left(m\pi f\right)}{{\left(m\pi \right)}^{2}}\sin^{2}\left(\frac{2\pi h}{\lambda }\right), & \mathrm{if}\;m=\pm 1,\pm 2\cdots \end{array}$$

### 2.2. Diffraction Efficiency of Segmented Phase Grating

**Figure 2:**
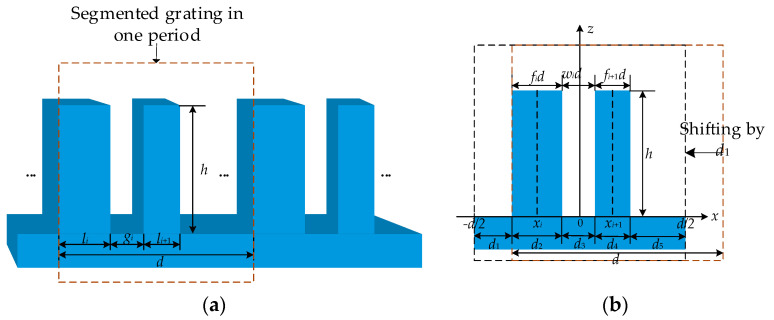
(**a**) The profile of a segmented phase grating; (**b**) a cross-section in one period.

Phase grating with more than one ridge in one period is called segmented phase grating [15]. Figure 2 illustrates the profile of segmented phase grating, where *f_i_d* is the width of the *i*^th^ ridge, *x_i_* is the center position of the *i*^th^ ridge, and *w_i_d* is the width of the *i*^th^ groove. In the following sections, a superscript ” indicates the identity of segmented grating.

When the segmented phase grating contains two ridges in one period, the length of each part is *d_k_*, as shown in Figure 2b. According to Equation (3), the diffraction field $U_{m}^{d_{k}}$ of each part is obtained by integration, and the diffraction field $U_{m}^{\prime \prime }$ of the *m*^th^ order is given by:

(6) $$\begin{array}{ll} U_{m}^{\prime \prime } & =\sum_{k=1}^{5}U_{m}^{d_{k}}=U_{\left(m,i\right)}+U_{\left(m,i+1\right)}-\frac{1}{d}\int_{-d/2}^{d/2}\exp\left(-j2m\pi x/d\right)dx\\ & =\{\begin{array}{ll} U_{\left(0,i\right)}+U_{\left(0,i+1\right)}-1, & \mathrm{if}\;m=0\\ U_{\left(m,i\right)}+U_{\left(m,i+1\right)}, & \mathrm{if}\;m=\pm 1,\pm 2,\ldots \end{array} \end{array}$$

where *U*_(*m*,*i*)_ is the diffraction field of the *i*^th^ grating ridge which can be considered as a standard phase grating as defined in Section 2.1, *m* denotes the order number, and *i* represents the *i*^th^ grating ridge. Assuming the total number of ridges is *N*, $U_{m}^{\prime \prime }$ becomes:

(7) $$U_{m}^{\prime \prime }=\{\begin{array}{ll} \sum_{i=1}^{N}U_{\left(0,i\right)}-\left(N-1\right), & \mathrm{if}\;m=0\\ \sum_{i=1}^{N}U_{\left(m,i\right)}, & \mathrm{if}\;m=\pm 1,\pm 2,\ldots \end{array}$$

To obtain $U_{m}^{\prime \prime }$, *U*_(*m*,*i*)_ is calculated. According to Equation (4) and the Fourier shift theorem [33], *U*_(*m*,*i*)_ can be expressed as:

(8) $$U_{\left(m,i\right)}=\{\begin{array}{ll} r\left[1-f_{i}+f_{i}\exp\left(-j\frac{4\pi h}{\lambda }\right)\right], & \mathrm{if}\;m=0\\ r\frac{\sin\left(m\pi f_{i}\right)}{m\pi }\left[\exp\left(-j\frac{4\pi h}{\lambda }\right)-1\right]\exp\left(-j\frac{2m\pi }{d}x_{i}\right), & \mathrm{if}\;m=\pm 1,\pm 2\cdots \end{array}$$

Substituting Equation (8) into Equation (7) yields:

(9) $$U_{m}^{\prime \prime }=\{\begin{array}{ll} r\left\{1+\sum_{i=1}^{N}\left[f_{i}\exp\left(-j\frac{4\pi h}{\lambda }\right)-f_{i}\right]\right\}, & \mathrm{if}\;m=0\\ r\sum_{i=1}^{N}\frac{\sin\left(m\pi f_{i}\right)}{m\pi }\left[\exp\left(-j\frac{4\pi h}{\lambda }\right)-1\right]\exp\left(-j\frac{2m\pi }{d}x_{i}\right), & \mathrm{if}\;m=\pm 1,\pm 2\cdots \end{array}$$

Hence, the diffraction efficiency $\eta_{m}^{\prime \prime }$ of segmented phase grating is given by:

(10) $$\eta_{m}^{\prime \prime }=\{\begin{array}{ll} r^{2}\left[1-4\sin^{2}\left(\frac{2\pi }{\lambda }h\right)\left(1-\sum_{i=1}^{N}f_{i}\right)\sum_{i=1}^{N}f_{i}\right], & \mathrm{if}\;m=0\\ r^{2}\frac{4}{{\left(m\pi \right)}^{2}}\sin^{2}\left(\frac{2\pi }{\lambda }h\right){\left|\sum_{i=1}^{N}\sin\left(m\pi f_{i}\right)\exp\left(-j\frac{2m\pi }{d}x_{i}\right)\right|}^{2}, & \mathrm{if}\;m=\pm 1,\pm 2\cdots \end{array}$$

## 3. Method of Segmented Phase Grating Design

### 3.1. Eliminating the Zeroth Order and Improving Efficiency of Odd Orders

**Figure 3:**
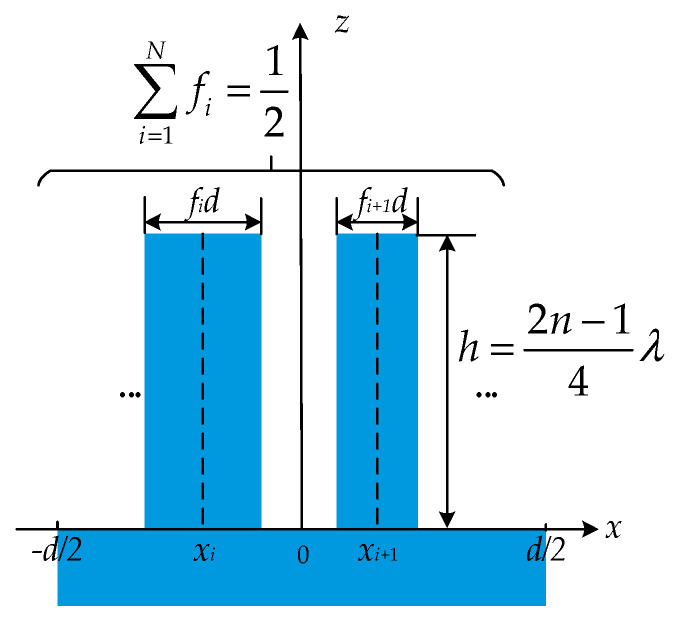
Segmented grating structure with the zeroth order eliminated.

To eliminate the diffraction light of the zeroth order, from Equation (9), the real and imaginary parts of the diffraction field $U_{0}^{\prime \prime }$ of the zeroth order should be zero. Hence, *h* = (2*n* − 1)*λ*/4 and $\sum_{i=1}^{N}f_{i}=0.5$, and they define a substantial structure, as shown in Figure 3. It demonstrates a segmented grating structure with the zeroth order eliminated.

**Figure 4:**
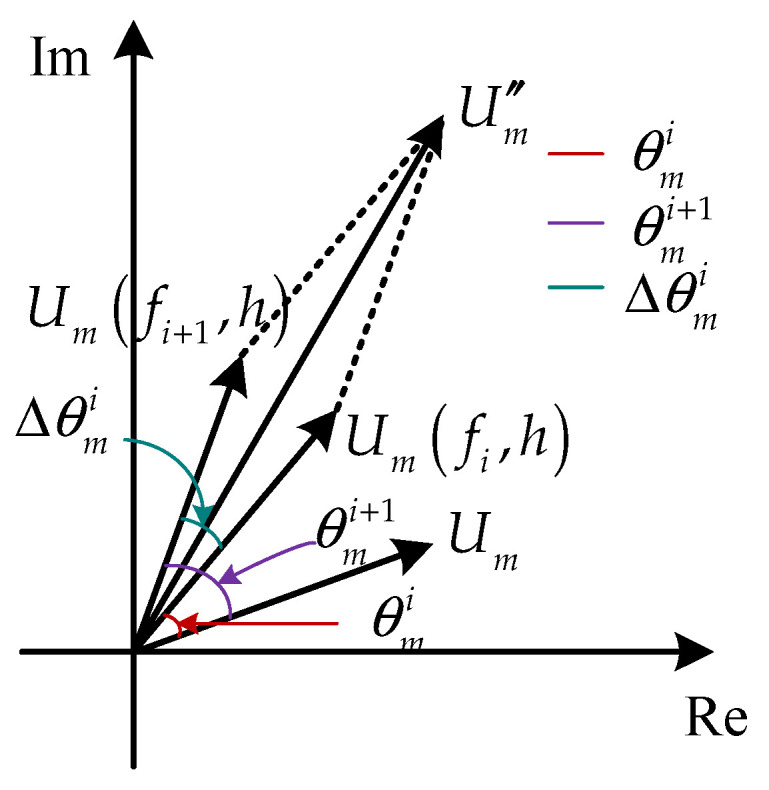
Schematic diagram of diffraction field vector superposition of segmented phase grating.

Except for the zeroth order, according to Equation (7), the diffraction field $U_{m}^{\prime \prime }$ can be taken as the sum vector of the diffraction fields *U*_(*m*,*i*)_ with different phase angles, as shown in Figure 4. According to Equation (8) and Equation (4), *U*_(*m*,*i*)_ is obtained by rotating *U_m_* through an angle $\theta_{m}^{i}=2m\pi x_{i}/d$. The angle $\Delta \theta_{m}^{i}$ between *U*_(*m*,*i*)_ and *U*_(*m*,*i*+1)_ is:

(11) $$\Delta \theta_{m}^{i}=\frac{2m\pi }{d}\left(x_{i+1}-x_{i}\right),$$

Hence, to improve the diffraction efficiency $\eta_{m}^{\prime \prime }$, *U_m_* needs to have the maximum amplitude and all *U*_(*m*,*i*)_ have the same direction. 

**Figure 5:**
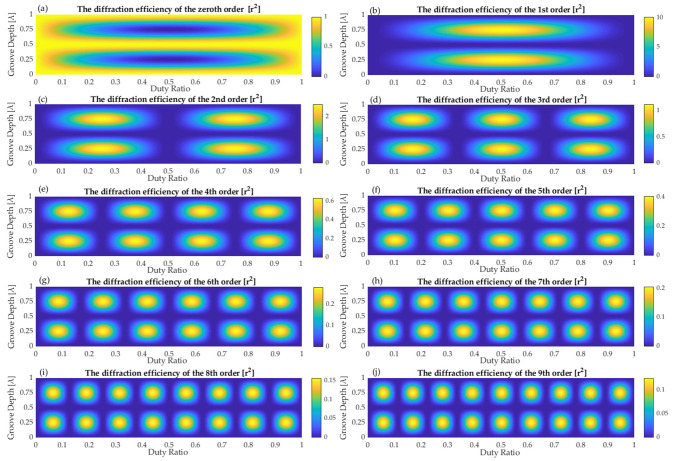
Diffraction efficiency of standard grating as a function of duty cycle and groove depth at diffraction orders from 0 to 9.

Firstly, conditions are analyzed when *U_m_* has the maximum amplitude based on Equations (4) and (5). Figure 5 illustrates the diffraction efficiency *η_m_* as a function of duty cycle *f* and groove depth *h*. As shown in Figure 5a–j, when *h* is a multiple of a half wavelength, the diffraction efficiency *η*_0_ of the zeroth order is *r*^2^, and the diffraction efficiency *η_m_* of other orders is equal to zero. When the product of *m* and *f* is an integer, the *m*^th^ order is absent. For example, when *f* is 1/5, *η*_5_ = 0. When *h* and *f* meet the following conditions at the same time:

(12) $$h=\frac{2n-1}{4}\lambda ,\;\mathrm{if}\;n=1,\;2,\cdots$$

(13) $$mf=n-\frac{1}{2},\;\mathrm{if}\;n=1,\;2,\cdots$$

*U_m_* has the maximum amplitude as:

(14) $$\left|U_{m}^{\max}\right|=2r/\left(m\pi \right),$$

From Equation (13), the value sin(*mf_i_*π) may be positive or negative. These two cases will be analyzed to investigate the influence of the sign of the sin(*mf_i_*π) value on the groove width *g_i_*.

When the signs of the sin(*mf_i_*π) and sin(*mf_i_*_+1_π) values are the same, to make directions of *U*_(*m*,*i*)_ and *U*_(*m*,*i*+1)_ consistent, $\Delta \theta_{m}^{i}$ must be:

(15) $$\Delta \theta_{m}^{i}=\frac{2m\pi }{d}\left(x_{i+1}-x_{i}\right)=2n\pi ,$$

The distance Δ*x_i_* between the *i*^th^ and (*i*+1)^th^ ridges should be:

(16) $$\Delta x_{i}=x_{i+1}-x_{i}=\frac{n}{m}d,$$

As shown in Figure 2, Δ*x_i_* is defined by the ridge width *l_i_* and the groove width *g_i_*. In this case, according to Equations (13) and (16), the groove width *g_i_* should be (2*n*–1)*d*/(2*m*). For example, for the 5th order, when both of *f_i_ and f_i_*_+1_ are 1/5, *g_i_* should be (2*n*–1)*d*/10 to ensure that *U*_(*m*,*i*)_ and *U*_(*m*,*i*+1)_ have the same direction.

When the signs of the sin(*mf_i_*π) and sin(*mf_i_*_+1_π) values are opposite, to make directions of *U*_(*m*,*i*)_ and *U*_(*m*,*i*+1)_ consistent, $\Delta \theta_{m}^{i}$ must be:

(17) $$\Delta \theta_{m}^{i}=\frac{2m\pi }{d}\left(x_{i+1}-x_{i}\right)=\left(2n-1\right)\pi ,$$

The distance Δ*x_i_* between the *i*^th^ and (*i*+1)^th^ ridges should be:

(18) $$\Delta x_{i}=x_{i+1}-x_{i}=\frac{2n-1}{2m}d,$$

In this case, according to Equations (13) and (16), the groove width *g_i_* should also be (2*n*–1)*d*/(2*m*). For example, for the 5th order, when *f_i_* and *f_i_*_+1_ are 1/5 and 3/5, respectively, *g_i_* should also be (2*n*−1)*d*/10 to ensure that *U*_(*m*,*i*)_ and *U*_(*m*,*i*+1)_ have the same direction. 

Whether the signs of the sin(*mf_i_*π) and sin(*mf_i_*_+1_π) values have the same or opposite sign, *g_i_* should be (2*n*−1)*d*/(2*m*) to make directions of *U*_(*m*,*i*)_ and *U*_(*m*,*i*+1)_ consistent. Thus, we obtain:

(19) $$\left|\sum_{i=1}^{N}\sin\left(m\pi f_{i}\right)\exp\left(-j\frac{2m\pi }{d}x_{i}\right)\right|=N,$$

Substituting Equations (12) and (19) into Equation (10), the enhanced diffraction efficiency $\eta_{m}^{\prime \prime }$ of the *m*^th^ order is expressed as:

(20) $$\eta_{m}^{\prime \prime }=r^{2}{\left(\frac{2N}{m\pi }\right)}^{2},$$

**Figure 6:**
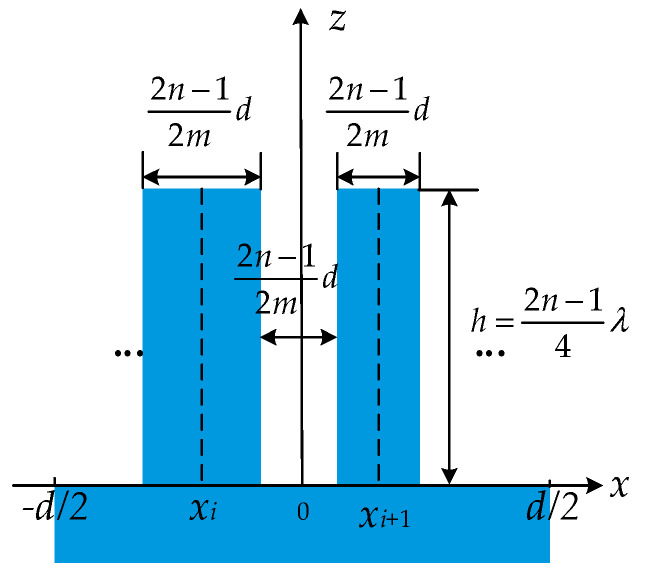
Segmented grating structure with the odd order m enhanced.

Figure 6 illustrates a segmented grating structure with high odd order *m* enhanced. 

### 3.2. Eliminating Diffraction of Even Orders

**Figure 7:**
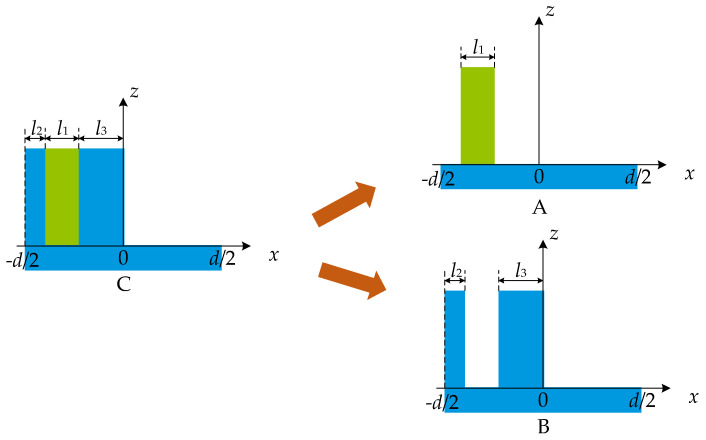
Grating C is divided into grating A and grating B.

When increasing the efficiency of a certain odd order, the efficiency of even orders is never zero. As shown in Figure 7, grating C with duty cycle *f* of 0.5 is composed of grating A and grating B. According to Equation (7), the diffraction field $U_{m}^{C}$ of grating C can be expressed as:

(21) $$U_{m}^{C}=U_{m}^{A}+U_{m}^{B},$$

where $U_{m}^{A}$ is the diffraction field of the *m^th^* order of grating A, $U_{m}^{B}$ is the diffraction field of the *m^th^* order of grating B, and then *m* cannot be zero. According to Equation (4), since *f* of grating C is 0.5, the diffraction field $U_{2m}^{C}$ is identically zero. Therefore, from Equation (21), the relationship between the diffraction field of grating A and grating B at even orders is given by:

(22) $$U_{2m}^{A}=-U_{2m}^{B},$$

**Figure 8:**
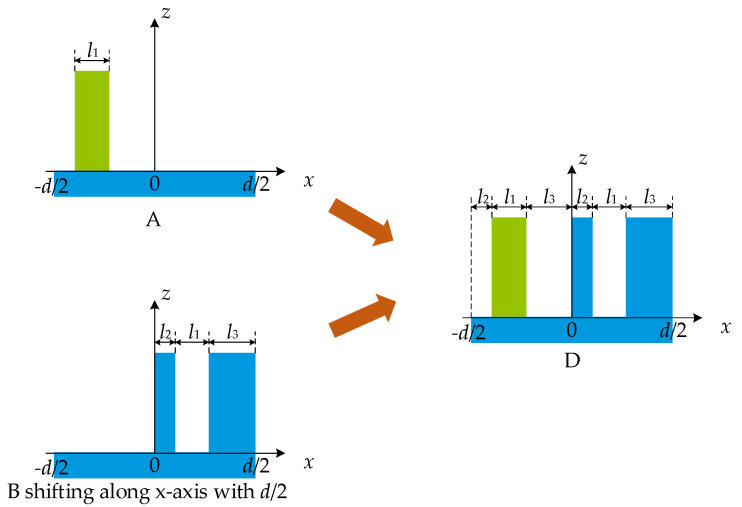
Grating D consists of grating A and grating B shifted along the *x*-axis with d/2.

As shown in Figure 8, grating D consists of grating A and grating B shifted along the *x*-axis with *d*/2. From Equation (9), the diffraction field $U_{m}^{D}$ can be expressed as:

(23) $$U_{m}^{D}=U_{m}^{A}+U_{m}^{B}\exp\left(-j\frac{2m\pi }{d}\times \frac{d}{2}\right),$$

Substituting Equation (22) into Equation (23) yields:

(24) $$U_{2m}^{D}=0,$$

Hence, when the segmented grating is divided into two parts in one period as grating A and grating B defined in Figure 8, even orders are eliminated. The conclusion is the same as the one obtained by Morrison with a different starting point [31,32]. In this work, the conclusion is derived from the diffraction field of the grating with a duty cycle of 0.5. Morrison derived it from the diffraction field of the Dammann grating. Hence, the conclusions are not new, but strongly supported by each other, and both further prove the practical value of the classical theory.

**Figure 9:**
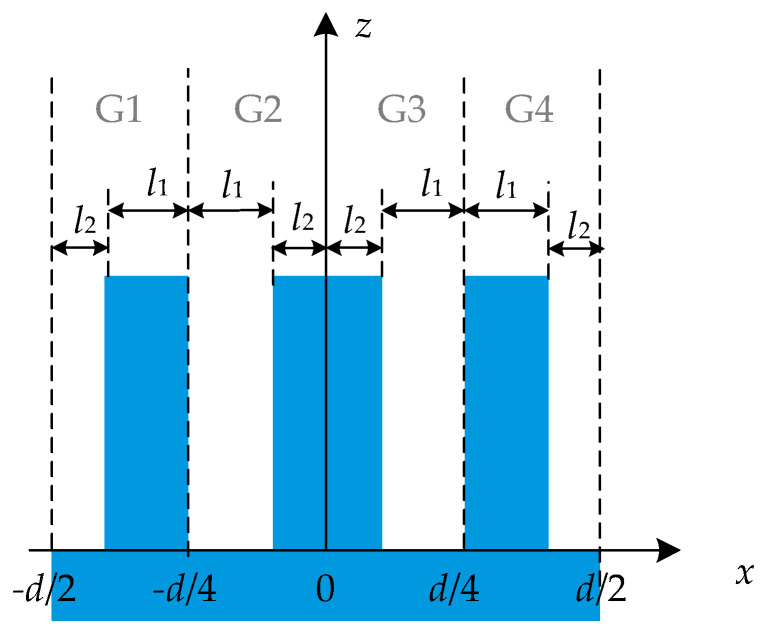
Segmented grating structure with even orders eliminated.

These two structures are defined as complementary to each other in this paper. Furthermore, the two parts must be symmetrical to each other according to the principle of alignment technology [1]. Therefore, the two parts can be divided into four parts by two lines *x* = ±*d*/4, as shown in Figure 9. G1 and G2 are complementary with respect to *x* = –*d*/4. Similarly, G3 and G4 are complementary with respect to *x* = *d*/4. G2 and G3 are symmetric around the *z*-axis, and so are G1 and G4.

**Figure 10:**
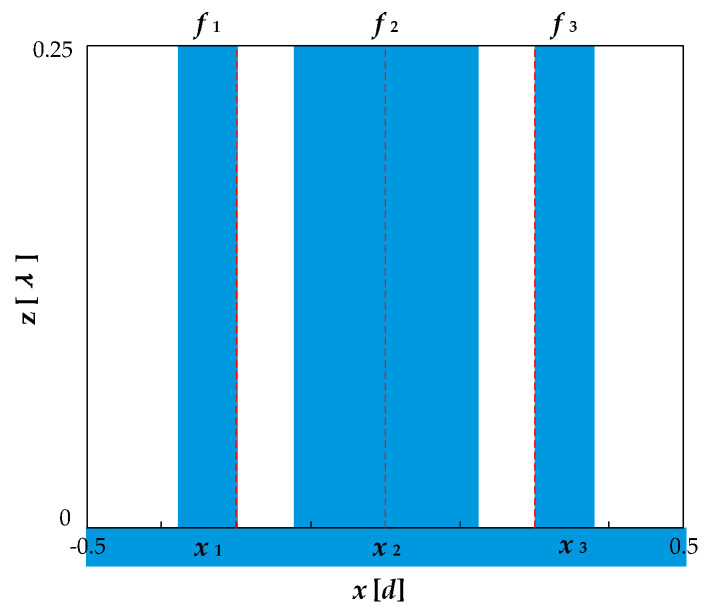
Grating structure with even diffraction orders eliminated when *n* = 3.

When the number of ridges *N* = 3 and the groove depth *h* = *λ*/4, the grating structure is shown in Figure 10, and its structural parameters satisfy the following requirements.

(25) $$\{\begin{array}{l} f_{2}=0.5-2f_{1},\;f_{3}=f_{1};\\ x_{1}=-0.25-f_{1}/2,\;x_{2}=0,\;x_{3}=-x_{1}; \end{array}$$

According to Equation (10), the diffraction efficiency is expressed as:

(26) $$\eta_{m}^{\prime \prime }=\{\begin{array}{ll} 0, & m=0,\pm 2,\pm 4\cdots \\ \frac{4r^{2}}{{\left(m\pi \right)}^{2}}{\left|1-4\sin^{2}\left(m\pi f_{1}\right)\right|}^{2}, & m=\pm 1,\pm 3,\pm 5\cdots \end{array}$$

**Figure 11:**
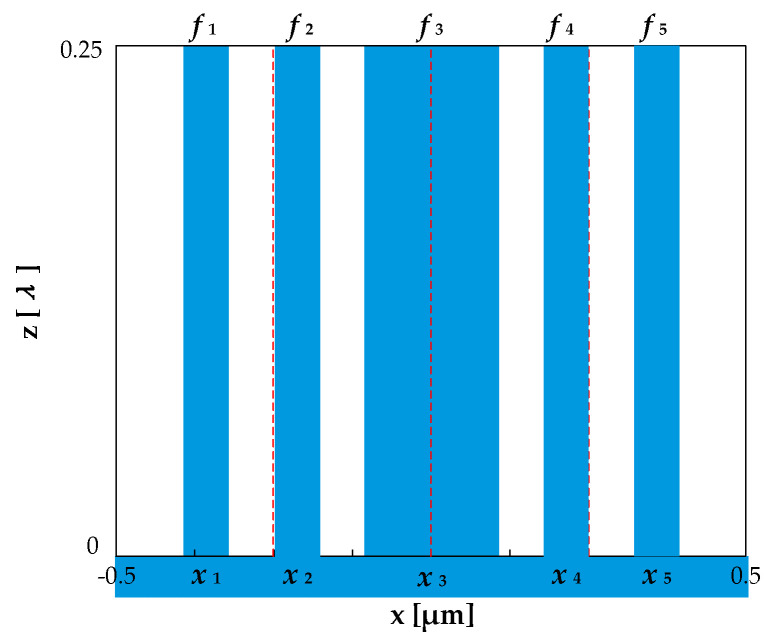
Grating structure with even diffraction orders eliminated when *n* = 5.

When *N* = 5 and *h* = *λ*/4, the grating structure is shown in Figure 11, and its structural parameters satisfy the following requirements.

(27) $$\{\begin{array}{l} f_{3}=0.5-2\times \left(f_{1}+f_{2}\right),\;f_{4}=f_{1},\;f_{5}=f_{2};\\ x_{1}=-0.25-f_{2}-f_{1}/2,\;x_{2}=-0.25+f_{2}/2,\;x_{3}=0,\;x_{4}=-x_{1},\;x_{5}=-x_{2}; \end{array}$$

According to Equation (10), the diffraction efficiency is expressed as:

(28) $$\eta_{m}^{\prime \prime }=\{\begin{array}{ll} 0, & m=0,\pm 2,\pm 4\cdots \\ \frac{4r^{2}}{{\left(m\pi \right)}^{2}}{\left|1-4\sin\left(m\pi f_{1}\right)\sin\left[m\pi \left(f_{1}+2f_{2}\right)\right]\right|}^{2}, & m=\pm 1,\pm 3,\pm 5\cdots \end{array}$$

### 3.3. Optimizing the Groove Depth

All previous cases are discussed in terms of one illumination wavelength *λ*. In this section, multiple wavelengths {*λ*_1_, …, *λ_n_*} are considered. When there is only one illumination wavelength *λ*, groove depth *h* should be equal to *λ*/4 in order to reduce the diffraction efficiency $\eta_{0}^{\prime \prime }$ of the zeroth order and enhance the diffraction efficiency $\eta_{m}^{\prime \prime }$ of the odd order *m*. However, there is no *h* that can satisfy this for all wavelengths at once. On the other hand, from Equations (13) and (16), ridge width *l*, groove width *g* are not affected by the multiple wavelengths. Here, we introduce the concept of average diffraction efficiency over different wavelengths which are to be minimized as ${\bar{\eta }}_{0}^{\prime \prime }$ and to be maximized as ${\bar{\eta }}_{m}^{\prime \prime }$ in order to get reasonable *h* overall wavelengths. A further discussion on the average efficiency for more than on odd orders will be addressed in much more detail in our future work.

When *l* and *g* meet enhancement and elimination requirements (Equations (20) and (24)), we call the grating enhanced grating. In this case, from Equation (10), ${\bar{\eta }}_{0}^{\prime \prime }$ and ${\bar{\eta }}_{m}^{\prime \prime }$ can be expressed as:

(29) $${\bar{\eta }}_{0}^{\prime \prime }=\frac{1}{4}\sum_{i=1}^{n}r_{i}{}^{2}\cos^{2}\left(\frac{2\pi h}{\lambda_{i}}\right),$$

(30) $${\bar{\eta }}_{m}^{\prime \prime }=\frac{1}{4}\sum_{i=1}^{n}r_{i}{}^{2}\sin^{2}\left(\frac{2\pi h}{\lambda_{i}}\right){\left|\sum_{i=1}^{N}\frac{\sin\left(m\pi f_{i}\right)}{m\pi }\exp\left(-j\frac{2m\pi }{d}x_{i}\right)\right|}^{2},$$

where *r_i_* is the reflection coefficient of grating illuminated by the wavelength *λ_i_*, *n* is the number of wavelengths. The derivatives of ${\bar{\eta }}_{0}^{\prime \prime }$ and ${\bar{\eta }}_{m}^{\prime \prime }$ with respect to *h* are given by:

(31) $$\frac{\partial {\bar{\eta }}_{0}^{\prime \prime }}{\partial h}=-\frac{\pi }{2}\sum_{i=1}^{n}\frac{r_{i}{}^{2}}{\lambda_{i}}\sin\left(\frac{4\pi h}{\lambda_{i}}\right),$$

(32) $$\frac{\partial {\bar{\eta }}_{m}^{\prime \prime }}{\partial h}=\frac{\pi }{2}\sum_{i=1}^{n}\frac{r_{i}{}^{2}}{\lambda_{i}}\sin\left(\frac{4\pi h}{\lambda_{i}}\right){\left|\sum_{i=1}^{N}\frac{\sin\left(m\pi f_{i}\right)}{m\pi }\exp\left(-j\frac{2m\pi }{d}x_{i}\right)\right|}^{2},$$

The optimal groove depth *h* is defined by:

(33) $$g\left(h\right)=\sum_{i=1}^{n}\frac{r_{i}{}^{2}}{\lambda_{i}}\sin\left(\frac{4\pi h}{\lambda_{i}}\right)=0,$$

**Figure 12:**
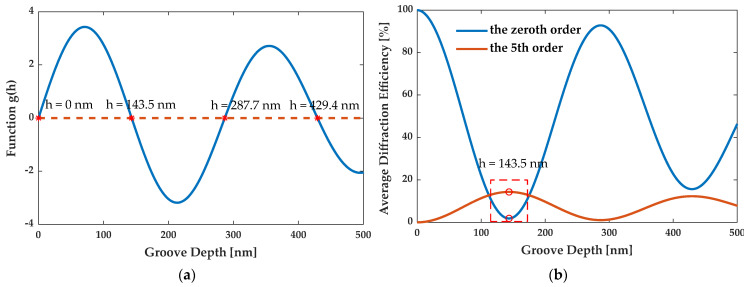
(**a**) The critical points of the groove depth h. (**b**) The average diffraction efficiency of the zeroth and 5th order as a function of the groove depth for the 5th order enhanced grating with three segmented ridges.

Figure 12a illustrates that if *h* is less than 500 nm and *r_i_* is equal to 1, there are four critical points at *h* = 0 nm, 143.5 nm, 287.7 nm, and 429.4 nm, for wavelengths of both 532 nm and 633 nm. Figure 12b shows ${\bar{\eta }}_{0}^{\prime \prime }$ and ${\bar{\eta }}_{5}^{\prime \prime }$ as a function of the parameter *h* for the 5th order enhanced grating with three segmented ridges. In Figure 12b, as ${\bar{\eta }}_{0}^{\prime \prime }$ increases with *h*, ${\bar{\eta }}_{5}^{\prime \prime }$ decreases with *h*. Conversely, as ${\bar{\eta }}_{0}^{\prime \prime }$ decreases with *h*, ${\bar{\eta }}_{5}^{\prime \prime }$ increases with *h*. When *h* = 143.5 nm, ${\bar{\eta }}_{0}^{\prime \prime }$ reaches the minimum in the validate scope, while ${\bar{\eta }}_{5}^{\prime \prime }$ reaches the maximum in the scope. Hence, *h* should be 143.5 nm at the illumination wavelengths of 532 nm and 633 nm.

### 3.4. Design Process of Segmented Grating

**Figure 13:**
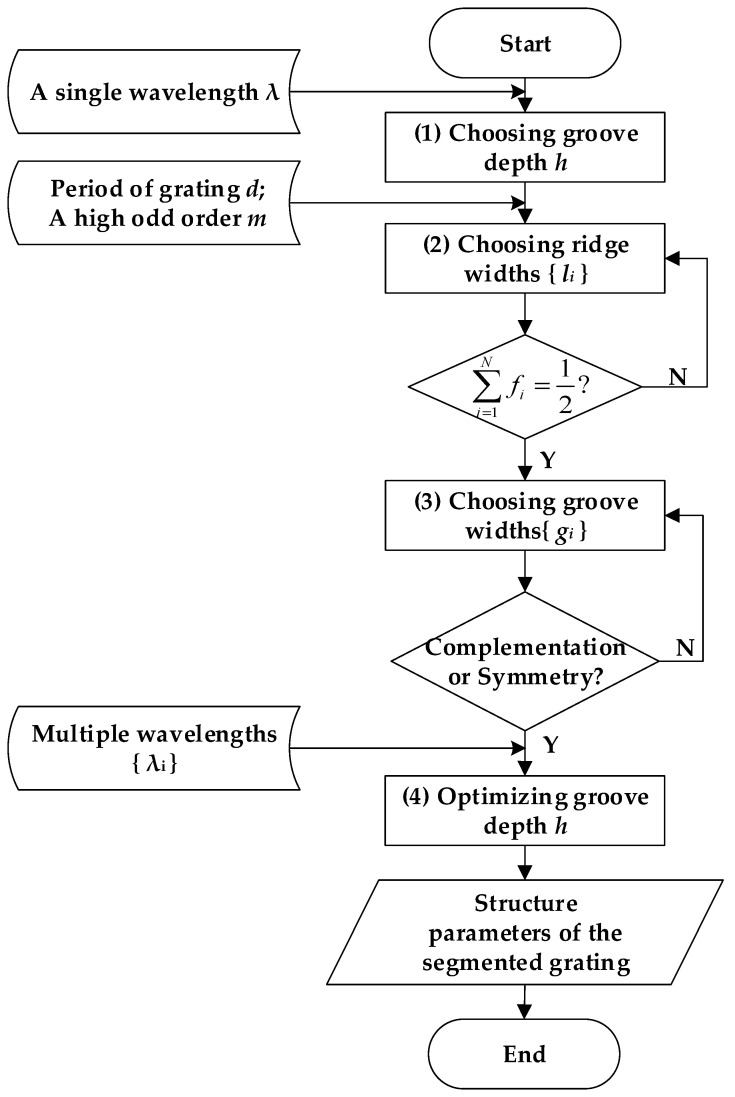
Flow chart of design method of segmented grating.

As a summary of the previous sections, Figure 13 describes the design process of a segmented grating with enhanced H-DLO as well as reduced DLZE. The detailed steps are illustrated as follows:

1. Choosing *groove depth h*: For a single wavelength λ, the groove depth *h* should be an odd multiple of the quarter wavelength to eliminate the zeroth order and to have the diffraction efficiency of other orders enhanced.
2. *Choosing* ridge *width* {*l_i_*}: In the case of the high odd order *m* and the grating period *d*, the ridge width *l_i_* should be (2*n*–1)*d*/(2*m*) to improve the diffraction efficiency of the order *m*. Furthermore, the sum of all ridge widths should be half of the grating period to satisfy the requirement of the zeroth order elimination.
3. *Choosing groove* width {*g_i_*}: The groove width *g_i_* should be (2*n*–1)*d*/(2*m*) to enhance the diffraction efficiency of the odd order *m*. In addition, to eliminate even orders, the groove widths *g_i_* should make the segmented grating structure complementary and symmetrical about the *z*-axis.
4. *Optimizing groove depth h*: For multiple wavelengths {λ_1_, …, λ*_n_*}, the groove depth *h* is optimized to decrease the average diffraction efficiency of the zeroth order. The optimal groove depth is defined by the material of the grating and illumination wavelengths. Finally, the structure of the segmented grating is obtained for all the requirements.

## 4. Designed Examples and Verification

Using the proposed method, as shown in Figure 13, we designed the grating with the 5th order enhanced and the grating with the 7th order enhanced. Meanwhile, the RCWA method was applied to calculate the efficiency of these segmented gratings to verify our design results. In the simulation, silicon was chosen as the material of the grating, and the period was defined as 16 μm. When the illumination wavelength is 633 nm and the light is perpendicular to the grating surface, the refractive index of silicon is 3.88 and the refractive index of the peripheral material is 1. Hence, the reflectance of the grating is 34.83% at 633 nm.

### 4.1. Comparison with RCWA Method

**Figure 14:**
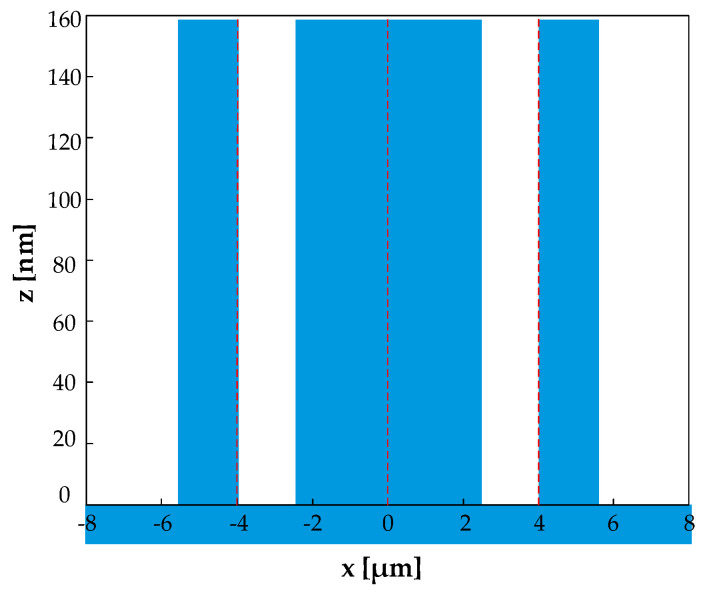
The structure of AH53_opt and AH53_opt’ in one period.

For the 5th order, AH53_opt was designed by the proposed method, and AH53_opt’ was obtained by VirtualLab Fusion software (RCWA). Table 1 compares the structure parameters of AH53_opt and AH53_opt’. Obviously, AH53_opt and AH53_opt’ have the same parameters. Table 1 describes the structure for both AH53_opt and AH53_opt’. Figure 14 illustrates the structure of the AH52_opt and AH53_opt’ in one period.

During the design process, four parameters needed to be optimized simultaneously in RCWA. At the same time, the target objective was to minimize $\eta_{0}^{\prime \prime }$ and $\eta_{2m}^{\prime \prime }$, and maximize $\eta_{5}^{\prime \prime }$. Even with a 32-core processor, the multi-objective optimization took three days to obtain satisfactory results. With the proposed method, it only took two hours to obtain the structure of Figure 14. Hence, the proposed method greatly improved the design efficiency. 

### 4.2. The 5th Order Enhancement Case

**Figure 15:**
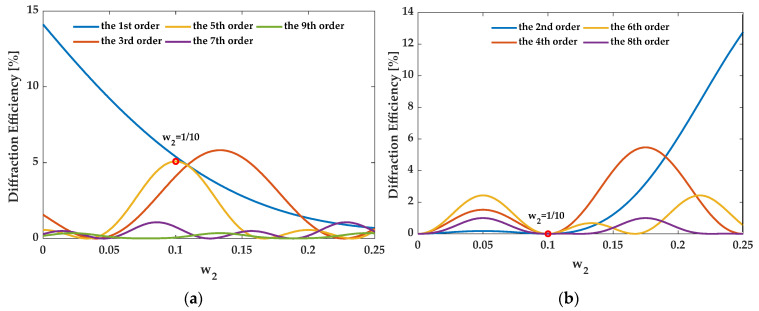
The diffraction efficiency of orders as a function of *w*_2_. (**a**) The odd orders, (**b**) the even orders.

**Figure 16:**
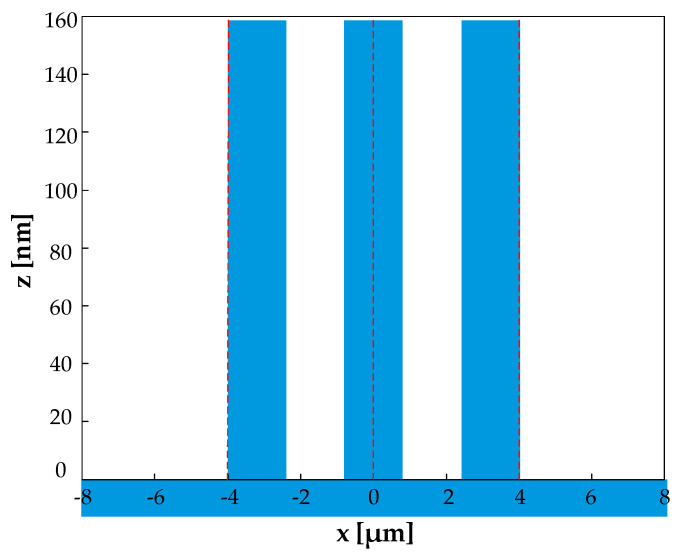
The structure of AH53 in one period.

For the 5th order enhancement, when the structure satisfies eliminating the zeroth order, {*f_i_*} should be {1/10, 3/10, 1/10}. Figure 15 illustrates that when {*f_i_*} is {1/10, 3/10, 1/10}, the diffraction efficiency is a function of *w*_2_ for orders 1–9. Structure AH53_opt (*w*_2_ = 1/10) enhances the 5th order and eliminates even orders. AH53_opt was compared to the equally segmented grating AH53 [15]. Table 2 compares the structure parameters of AH53 and AH53_opt. Figure 16 illustrates the profiles of AH53 with a period of 16 μm [15]. Table 3 compares the diffraction efficiency of them for the different orders 0–9. It can be found that all of their structure parameters satisfy the enhancement requirement of the 5th order, and they have the same number of segmented ridges *N*. Hence, both gratings have the same $\eta_{5}^{\prime \prime }$ of 5.0%. However, for AH53, the sum of all ridge widths is not half period, and the structure does not meet the elimination requirement of the zeroth and even orders. Therefore, $\eta_{0}^{\prime \prime }$ and $\eta_{2m}^{\prime \prime }$ are not zero. For AH53_opt, $\eta_{0}^{\prime \prime }$ and $\eta_{2m}^{\prime \prime }$ are identical to zero.

### 4.3. The 7th Order Enhancement Case

**Figure 17:**
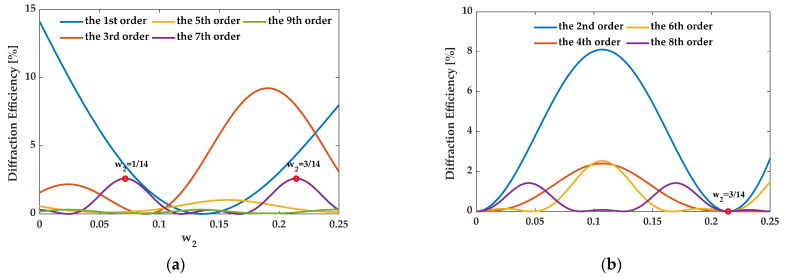
The diffraction efficiency of orders as a function of *w*_2_ when {*f_i_*} is {3/14, 1/14, 3/14}. (**a**) The odd orders, (**b**) the even orders.

**Figure 18:**
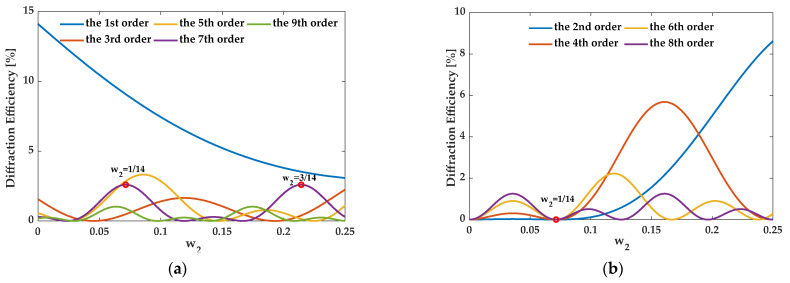
The diffraction efficiency of orders as a function of *w*_2_ when {*f_i_*} is {1/14, 5/14, 1/14}. (**a**) The odd orders, (**b**) the even orders.

**Figure 19:**
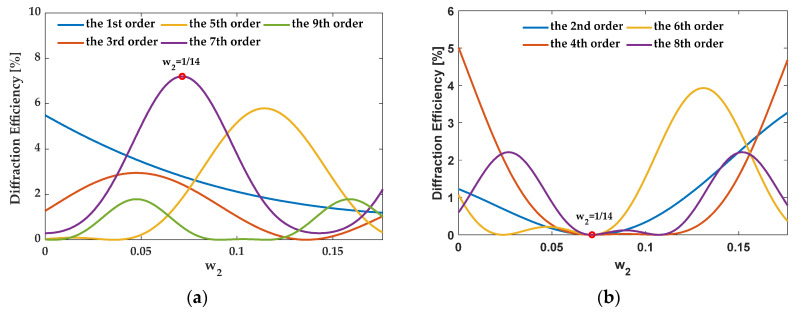
The diffraction efficiency as a function of *w*_2_ when {*f_i_*} is {1/14, 1/14, 3/14, 1/14, 1/14}. (**a**) The odd orders, (**b**) the even orders.

**Figure 20:**
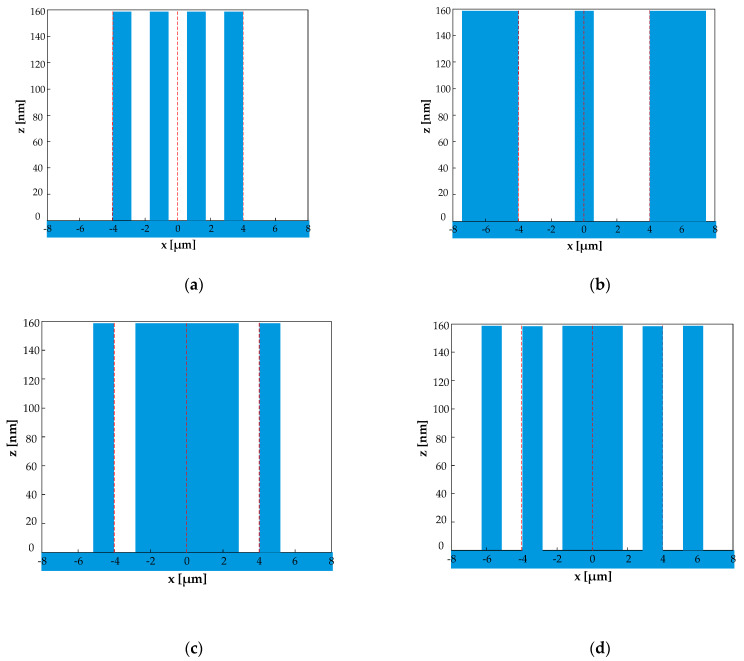
The structures of AH74 and AH74_opt in one period. (**a**) AH74, (**b**) AH74_opt1, (**c**) AH74_opt2, (**d**) AH74_opt3.

For the 7th order enhancement, when the structure satisfies eliminating the zeroth order, {*f_i_*} should be {3/14, 1/14, 3/14}, {1/14, 5/14, 1/14}, and {1/14, 1/14, 3/14, 1/14, 1/14}. In this case, Figure 17, Figure 18 and Figure 19 illustrate the diffraction efficiency as a function of *w*_2_ for orders 0–9. Three structures, AH74_opt1 (*w*_2_ = 3/14), AH74_opt2 (*w*_2_ = 1/14), and AH74_opt3 (*w*_2_ = 1/14), enhance the 7th order and eliminate even orders. Compared with AH74 [15], Table 4 lists the structure parameters of four gratings. Figure 20 shows the profiles of these gratings with a period of 16 μm. Table 5 compares the diffraction efficiency of these gratings for the different orders 0–9. The groove depth *h*, the ridge widths *l_i_*, and the groove widths *g_i_* of these gratings all satisfy the requirements of enhancing the 7th order. Hence, $\eta_{7}^{\prime \prime }$ depends on the number *N* of segmented ridges. AH74_opt3 has the maximum *N*, so AH74_opt3 has a maximum $\eta_{7}^{\prime \prime }$. Moreover, except for AH74, other structures all eliminate the zeroth and even orders. Therefore, for AH74_opt1, AH74_opt2, and AH74_opt3, $\eta_{0}^{\prime \prime }$ and $\eta_{2m}^{\prime \prime }$ are equal to zero, while $\eta_{0}^{\prime \prime }$ is up to 5.9% for AH74. Furthermore, AH74_opt2 has the highest $\eta_{5}^{\prime \prime }$ and $\eta_{9}^{\prime \prime }$, which are 28 and 9 times higher than AH74, respectively.

### 4.4. Multi-Wavelength Case

**Figure 21:**
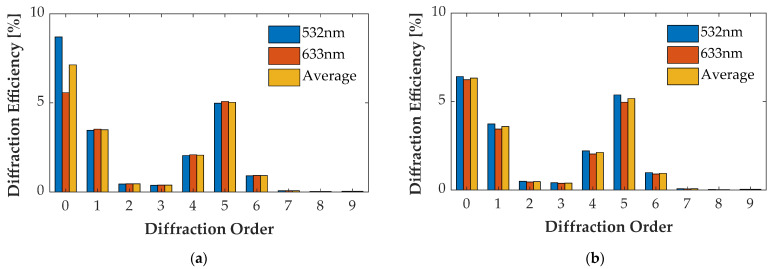
Diffraction efficiency of AH53. (**a**) AH53 with the groove depth of 158.25 nm; (**b**) AH53 with the groove depth of 143 nm.

**Figure 22:**
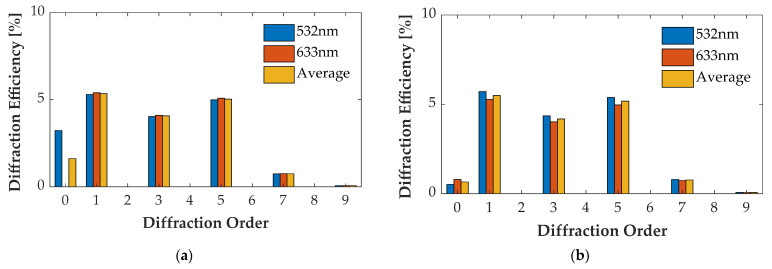
Diffraction efficiency of AH53_opt. (**a**) AH53_opt with the groove depth of 158.25 nm; (**b**) AH53_opt with the groove depth of 143 nm.

**Figure 23:**
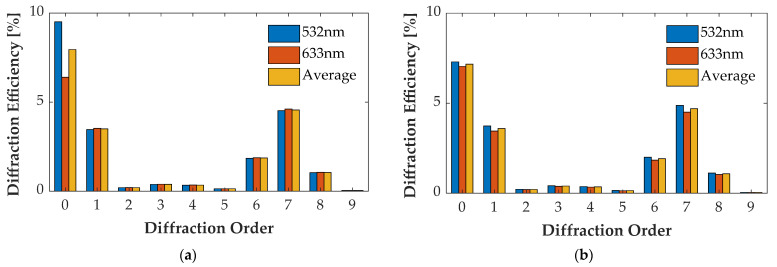
Diffraction efficiency of AH74. (**a**) AH74 with the groove depth of 158.25 nm; (**b**) AH74 with the groove depth of 143 nm.

**Figure 24:**
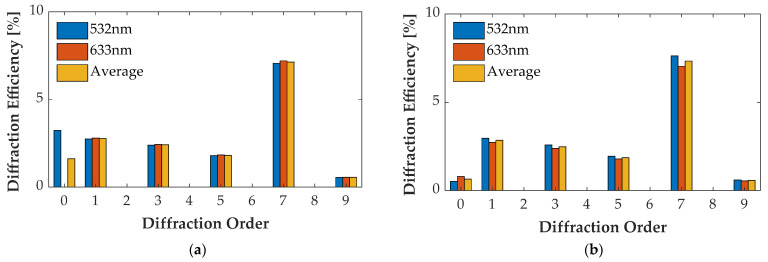
Diffraction efficiency of AH74_opt3. (**a**) AH74_opt3 with the groove depth of 158.25 nm; (**b**) AH74_opt3 with the groove depth of 143 nm.

For the wavelength of 532 nm and 633 nm, optimizing groove depth *h* decreased ${\bar{\eta }}_{0}^{\prime \prime }$ and increased ${\bar{\eta }}_{m}^{\prime \prime }$ of other orders. Figure 21a, Figure 22a, Figure 23a and Figure 24a illustrate the diffraction efficiency of AH53, AH53_opt, AH74, and AH74_opt3 with *h* = 158.25 nm for wavelengths 532 nm and 633 nm. According to the design method, *h* has a critical point which has the lowest ${\bar{\eta }}_{0}^{\prime \prime }$ and the largest ${\bar{\eta }}_{m}^{\prime \prime }$. The optimal *h* of AH53_opt and AH74_opt3 is equal to 143 nm. The optimal *h* is defined by the material of the grating and the illumination wavelength, and has nothing to do with the structures of these gratings according to (33). Figure 21b, Figure 22b, Figure 23b and Figure 24b illustrate the diffraction efficiency of AH53, AH53_opt, AH74, and AH74_opt3 with *h* = 143 nm. It can be found that ${\bar{\eta }}_{0}^{\prime \prime }$ dramatically decreases when *h* = 143 nm. Moreover, at the same time, ${\bar{\eta }}_{m}^{\prime \prime }$ increases to some extent. For AH53_opt and AH74_opt3, ${\bar{\eta }}_{m}^{\prime \prime }$ increases more than AH53 and AH74, and ${\bar{\eta }}_{2m}^{\prime \prime }$ are still equal to zero.

Thus far, one odd order has been considered as the major target for optimization, such as the 5th or the 7th order. In future work, optimizing over the 5th, the 7th, and the 9th orders simultaneously can be discussed in depth, especially for multi-wavelength cases.

### 4.5. Comparing the Design Time with RCWA

**Figure 25:**
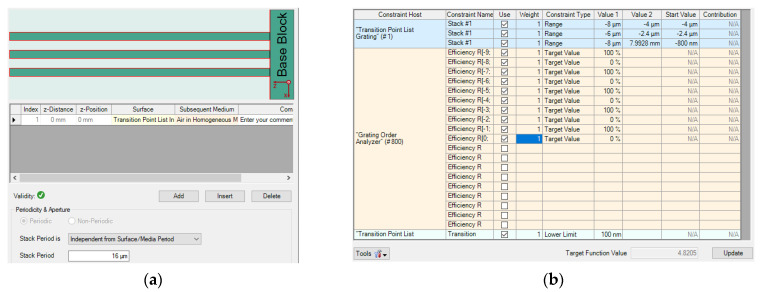
Optimized AH53. (**a**) Initial structure; (**b**) objective functions.

**Figure 26:**
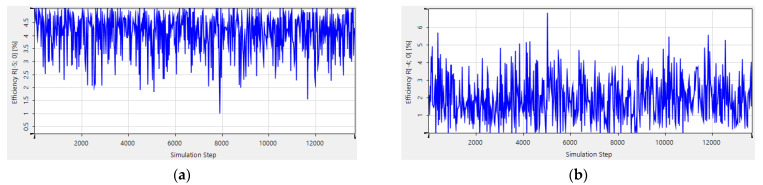
The diffraction efficiency after 13,000 iterations. (**a**) The 5th order; (**b**) the 4th order.

**Figure 27:**
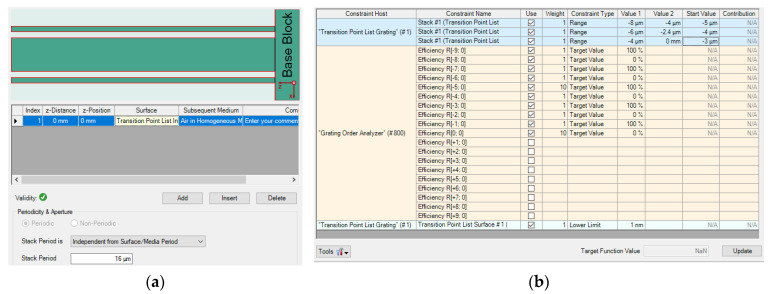
Optimized AH53. (**a**) Initial structure; (**b**) objective functions.

**Figure 28:**
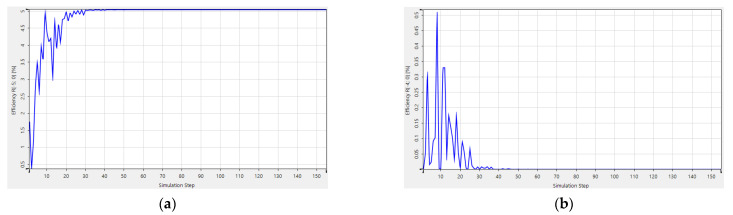
The diffraction efficiency after 150 iterations. (**a**) The 5th order; (**b**) the 4th order.

Multi-parameter optimization is quite sensitive to the initial conditions. There is always a risk that no structures are produced to meet the complex requirements. Besides that, it is a lengthy and costly process to calculate multiple variables simultaneously with a high-powered computing processor. For example, the starting point and the target function are demonstrated in Figure 25, and the structure of AH53_opt (designed in Section 4.1) is not able to be obtained by means of RCWA, as shown in Figure 26. After adjusting the parameters several times, the starting point and the target function are demonstrated in Figure 27, and the Ah53_opt structure is obtained, as shown in Figure 28. Table 6 summarizes the time when multi-parameter optimization is applied. It is worth mentioning that it took only 2 h to obtain AH53 by means of our method.

## 5. Conclusions

In this paper, we propose a design method for segmented phase grating with increasing intensity of the measurement signal and limited stray light to improve the SNR in a PGA sensing system. An analytical model has been constructed to describe the relationship between the diffraction field and the structure of the segmented grating. This model has provided insight into the diffraction theory of the segmented phase grating and has helped to find the most effective approach to design a required grating structure. Compared with numerical simulation methods, the simulation results demonstrate the effectiveness and efficiency of the proposed method. It also reduces the design time from several days to a few hours. By means of this method, a grating structure with the 5th order enhanced has been designed and analyzed, as well as three structures with the 7th order enhanced. Compared with an equally segmented grating structure, the diffraction efficiency of the diffraction light of the zeroth and the even orders (DLZE) has been dramatically inhibited while the diffraction efficiency of the diffraction light of the high odd orders (H-DLO) has been enhanced. These designed structures can be applied to alignment marks, which are widely used in PGA sensors and greatly improve the measurement accuracy. Furthermore, this design method can be applied to not only the alignment system but also other systems with grating-based measurement such as spectrometers, wavelength division multiplexing, visual display technology, etc.

## Figures and Tables

**Table 1:** The structure parameters of AH53_opt and AH53_opt’.

Item/Grating	AH53_opt	AH53_opt’
Groove depth *h* (nm)	158.25	158.25
Number of ridges *N*	3	3
Ridge width/Period {*f_i_*}	1/10, 3/10, 1/10	1/10, 3/10, 1/10
Sum ridge widths/Period *f*	1/2	1/2
Groove width/Period {*w_i_*}	1.5/10, 1/10, 1/10, 1.5/10	1.5/10, 1/10, 1/10, 1.5/10

**Table 2:** The structure parameters of AH53 and AH53_opt.

Item/Grating	AH53	AH53_opt	Design Requirements
Groove depth *h* (nm)	158.25	158.25	(2*n* − 1)λ/4
Number of ridges *N*	3	3	≥3
Ridge width/Period {*f_i_*}	1/10, 1/10, 1/10	1/10, 3/10, 1/10	(2*n* − 1)/10
Sum ridge widths/Period *f*	3/10	1/2	1/2
Groove width/Period {*w*_i_}	1/4, 1/10, 1/10, 1/4	1.5/10, 1/10, 1/10, 1.5/10	(2*n* − 1)/10

**Table 3:** Comparison the efficiency of each order for AH53 and AH53_opt.

Grating/Order	0	1	2	3	4	5	6	7	8	9
AH53	5.0%	3.8%	0.5%	0.4%	2.1%	5.0%	0.9%	0.1%	0.0%	0.0%
AH53_opt	0.0%	5.4%	0.0%	4.1%	0.0%	5.0%	0.0%	0.7%	0.0%	0.1%

**Table 4:** The structure parameters of AH74, AH74_opt1, AH74_opt2, AH74_opt3.

Item/Grating	AH74	AH74_opt1	AH74_opt2	AH74_opt3	Design Requirements
Groove depth *h* (nm)	158.25	158.25	158.25	158.25	(2*n* − 1)*λ*/4
Number of ridges *N*	4	3	3	5	≥3
Ridge width/Period {*f_i_*}	1/14, 1/14, 1/14, 1/14	3/14, 1/14, 3/14	1/14, 5/14, 1/14	1/14, 1/14, 3/14, 1/14, 1/14	(2*n* − 1)/14
Sum ridge widths/Period *f*	4/14	1/2	1/2	1/2	1/2
Groove width/Period {*w_i_*}	1/4, 1/14, 1/14, 1/14, 1/4	0.5/14, 3/14, 3/14, 0.5/14	2.5/14, 1/14, 1/14, 2.5/14	1.5/14, 1/14, 1/14, 1/14, 1/14, 1.5/14	(2*n* − 1)/14

**Table 5:** Comparison of the efficiency of each order for AH74, AH74_opt1, AH74_opt2, AH74_opt3.

Grating/Order	0	1	2	3	4	5	6	7	8	9
AH74	5.9%	3.7%	0.2%	0.4%	0.3%	0.1%	2.0%	4.6%	1.0%	0.1%
AH74_opt1	0.0%	4.4%	0.0%	7.9%	0.0%	0.4%	0.0%	2.6%	0.0%	0.1%
AH74_opt2	0.0%	9.1%	0.0%	0.5%	0.0%	2.8%	0.0%	2.6%	0.0%	0.9%
AH74_opt3	0.0%	2.8%	0.0%	2.4%	0.0%	1.8%	0.0%	7.2%	0.0%	0.6%

**Table 6:** The time spent designing AH53_opt’ with VirtualLab Fusion software.

Item	Number of Times to Change the Initial Value	Average Number of Iterations per Initial Value	Time per Iteration (S)	Total Time (Days)
Value	20	5000	3	3.5

## Data Availability

Not applicable.
